# Protection of European domestic pigs from virulent African isolates of African swine fever virus by experimental immunisation

**DOI:** 10.1016/j.vaccine.2011.04.052

**Published:** 2011-06-20

**Authors:** Katherine King, Dave Chapman, Jordi M. Argilaguet, Emma Fishbourne, Evelyne Hutet, Roland Cariolet, Geoff Hutchings, Christopher A.L. Oura, Christopher L. Netherton, Katy Moffat, Geraldine Taylor, Marie-Frederique Le Potier, Linda K. Dixon, Haru-H. Takamatsu

**Affiliations:** a Institute for Animal Health Pirbright Laboratory, Pirbright, Woking, Surrey GU24 0NF, UK; b Royal Veterinary College, University of London, Hatfield, Hertfordshire AL9 7TA, UK; c Centre de Recerca en Sanitat Animal, Campus de la UAB, Barcelona, Spain/Biologia de la Infecció, CEXS, Universitat Pompeu Fabra, Barcelona, Spain; d Anses, Laboratoire de Ploufragan, Unité Virologie Immunologie Porcines, Zoopôle Les Croix, B.P. 53, 22440 Ploufragan, France

**Keywords:** African swine fever, Asfarviridae, Pigs, Protection, Immunisation

## Abstract

African swine fever (ASF) is an acute haemorrhagic disease of domestic pigs for which there is currently no vaccine. We showed that experimental immunisation of pigs with the non-virulent OURT88/3 genotype I isolate from Portugal followed by the closely related virulent OURT88/1 genotype I isolate could confer protection against challenge with virulent isolates from Africa including the genotype I Benin 97/1 isolate and genotype X Uganda 1965 isolate. This immunisation strategy protected most pigs challenged with either Benin or Uganda from both disease and viraemia. Cross-protection was correlated with the ability of different ASFV isolates to stimulate immune lymphocytes from the OURT88/3 and OURT88/1 immunised pigs.

## 1 Introduction

African swine fever (ASF) is a highly contagious, haemorrhagic disease of pigs caused by a large, cytoplasmic, icosahedral DNA virus (ASFV) with a genome size of 170–193 kbp. Virulent isolates kill domestic pigs within 7–10 days of infection. In chronic cases ASF causes respiratory disorders and in some cases swelling around the leg joints and skin lesions. Domestic pigs can survive infection with less virulent isolates and in doing so can gain immunity to subsequent challenge with related virulent viruses [1–5].

ASF is endemic in many sub-Saharan African countries as well as in Sardinia. In 2007 ASF was introduced into Georgia and from there spread rapidly to neighbouring countries in the Trans Caucasus region, including Southern European Russia [6]. The virus has continued to spread through the Russian Federation and 18 federal subjects have reported outbreaks (OIE WAHID). Virus has also been isolated a number of times from wild boar in this region and the presence of ASF in this wildlife population is likely to make eradication more difficult [6].

Genotyping of ASFV isolates by partial sequencing of the B646L gene encoding the major capsid protein p72 has identified up to 22 genotypes [7,8]. Many of these are circulating in the long-established sylvatic cycle involving soft ticks of *Ornithodoros* spp. and warthogs in eastern and southern Africa. In many regions the isolates circulating in domestic pigs are genetically more similar.

Previous work has shown that pigs are protected from challenge with related virulent isolates following infection with natural low virulence isolates and with virus attenuated by passage in tissue culture or by deletion of genes involved in virulence [2,3,9,10]. Protection induced by the non-virulent OURT88/3 isolate was shown to require CD8^+^ T cells since depletion of these cells was shown to abrogate this protection [11]. Passive transfer of antibodies from pigs protected following infection with lower virulence isolates was also shown to protect naïve pigs from challenge with related virulent virus [12]. Although they are effective in inducing protection, there are safety issues related to the release of attenuated live vaccines. For example, following the introduction of ASF to Spain and Portugal in 1960, field isolate viruses were serially passed through primary bone marrow or blood macrophage cell cultures and then used to vaccinate pigs in Spain and Portugal. A substantial proportion of the half million pigs vaccinated in Portugal developed unacceptable post-vaccination reactions, including death [13]. In addition, a large number of carrier animals were generated, hindering subsequent attempts to eradicate the disease [14]. In the absence of a vaccine, control measures are currently limited to slaughter and the application of strict animal movement restriction policies.

Despite this early experience in Portugal and Spain, the prospect of developing successful attenuated vaccines have improved as substantial progress has been made in identifying ASFV genes involved in virulence and immune evasion and the complete coding sequences of a number of ASFV isolates are now available [15–17]. This information provides a route to the rational construction of attenuated ASFV vaccines. Currently knowledge of the antigens involved in protective immunity and the ability of isolates to confer cross-protection is limited. In this study we extended our previous work with an experimental ASFV vaccination strategy based on the non-virulent genotype I OURT88/3 isolate from Portugal. We confirmed that immunisation with this isolate followed by the virulent OURT88/1 isolate confers protection against challenge with two virulent isolates from Africa, one, Benin 97/1, from the same genotype I and the other, virulent Uganda 1965, from genotype X. We also show that the ability of different ASFV isolates to stimulate IFN-γ production from the immune pig lymphocytes correlates with the ability to induce cross-protection against different isolates. Thus this assay is useful to predict cross-protection and vaccine efficacy. These results suggest that ASFV vaccines which cross-protect more broadly could be produced, extending the possible use of a vaccination strategy.

## 2 Materials and methods

### 2.1 ASFV virus isolates

ASFV isolates used in this study have been described previously and included Portuguese isolates of ASFV, OURT88/3 (non-virulent, non-haemadsorbing, genotype I) and OURT88/1 (virulent, haemadsorbing, genotype I) [2], virulent Portuguese pig isolate Lisbon 57 (genotype I; [18]), moderately virulent Malta isolate Malta/78 (genotype I; [19]), virulent West African isolate Benin 97/1 (genotype I; [15]) virulent African isolates Uganda 1965 (genotype X; [20]) and Malawi Lil 20/1 (genotype VIII; [21]). Viruses were grown in primary porcine macrophage cultures and used after limited passage.

### 2.2 Experimental design of pig experiments

Pigs used in the first experiment (experiment 1) at IAH Pirbright Laboratory UK were cross-bred pigs, Large White and Landrace, of average weight 20 kg at the first immunisation. For the second experiment specific pathogen free (SPF) Large White pigs were used from Anses, Ploufragan, France, SPF facility and were of 15 kg average weight at the first immunisation (experiment 2). For the third experiment (experiment 3) carried out at Anses Ploufragan, France, Large White pigs were obtained from a local high health status farm and the average weight at the first immunisation was 11 kg. All pigs were maintained at high security facilities throughout the experiment. The first experiment at Pirbright was performed under Home Office licence PPL 70-6369. Experiments at Ploufragan were performed according to the animal welfare experimentation agreement given by the Direction des Services Vétérinaires des Côtes d’Armor (AFSSA registration number B-22-745-1), under the responsibility of Marie-Frédérique Le Potier (agreement number 22-17). Briefly, pigs were intramuscularly inoculated with 10^4^ TCID_50_ of non-virulent ASFV isolate OURT88/3 and boosted intramuscularly 3 weeks later with 10^4^ HAD_50_ of virulent ASFV isolate of OURT88/1. Pigs were then challenged 3 weeks later with 10^4^ HAD_50_ of either Benin 97/1 or virulent Uganda 1965 intramuscularly.

### 2.3 Clinical and pathological observations

ASFV-inoculated pigs were monitored for body temperature and other clinical symptoms and these were recorded and scored according to the clinical scoring system shown in Supplementary Table 1. Weight gain was also recorded in the experiments carried out at Ploufragan. All pigs were examined post-mortem either when the pigs died or at the termination of the experiments. Tissues were collected for further analysis.

### 2.4 ASFV detection

Peripheral blood was analysed at different days post-immunisation for the presence of ASFV by quantitative PCR (qPCR) as described previously [22]. Samples which tested positive by qPCR were further analysed by cytopathic and/or haemadsoption assay (HAD) using standard pig bone marrow cells in 96 well plate [23,24]. Spleen, tonsil, retropharyngeal and ileocaesal lymph nodes from post-mortem tissues were also analysed for the presence of ASFV by qPCR and HAD. Virus detected from tissue samples by qPCR was expressed as copy number per mg tissue and by HAD as HAD_50_.

### 2.5 Analysis of immune responses against ASFV

Development of T cell immune responses to ASFV after immunisation was analysed by IFN-γ ELISPOT and proliferation assays as described previously [25]. All ASFV isolates used as antigens for T cell assays were prepared by culture in porcine bone marrow cells, and ASFV titres were determined by qPCR [22] and adjusted to give the equivalent of 10^5^ HAD_50_/ml. Uninfected porcine bone marrow culture supernatants were used as negative control antigen.

The development of ASFV specific antibodies was analysed using a competition ASF ELISA kit (INGENASA PPA3 COMPPAC), and the antibody titre was expressed as log 2 dilution of end point which gives 50% competition.

## 3 Results

### 3.1 Protection of ASFV immunised pigs from challenge with virulent isolates

Three experiments were carried out in which pigs were immunised with the non-virulent Portuguese OURT88/3 genotype I isolate followed 3 weeks later by the closely related virulent Portuguese isolate OURT88/1 and then challenged 3 weeks later with either the West African genotype I isolate, Benin 97/1, or the genotype X virulent Uganda 1965 isolate. In the first experiment at Pirbright, 3 immunised pigs and 4 non-immune pigs were challenged with Benin 97/1. In the second experiment at Ploufragan, a total of 12 pigs were immunised and challenged with either Benin 97/1 or virulent Uganda 1965. Ten pigs were prepared as non-immune controls and challenged with either Benin 97/1 or virulent Uganda 1965. As a control for weight gain, an extra group of 5 pigs were included in this experiment. In the third experiment at Ploufragan, a group of 7 pigs were inoculated and 6 of these and 6 non-immunised pigs were challenged with Benin 97/1.

**Fig. 1:**
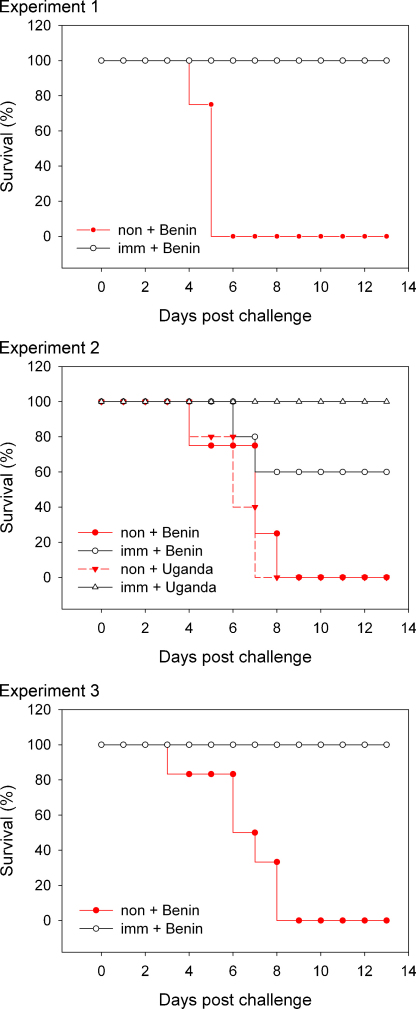
Summary results from three separate ASFV challenge/protection experiments. The *y*-axis shows the percentage of pigs which survived following challenge and the *x*-axis shows time post-challenge in days. Non-immune pig groups challenged with virulent ASFV are shown as red lines and the challenge virus strain is indicated as Benin for Benin 97/1, Uganda for virulent Uganda 1965. Immune pigs challenged are shown as black lines and are labelled imm + Benin for immunised pigs challenged with Benin 97/1 or imm + Uganda for immunised pigs challenged with virulent Uganda 1965.

**Fig. 2:**
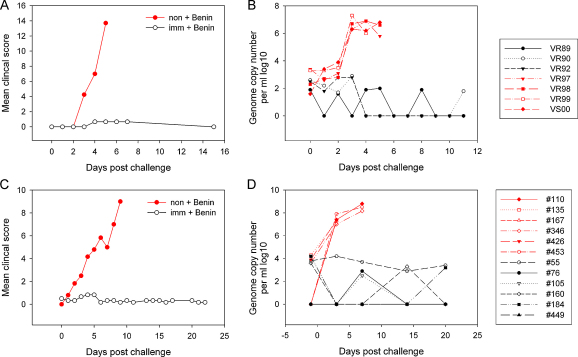
Clinical scores and viraemia from experiment 1 (A and B) and experiment 3 (C and D). Clinical scores for experiment 1 are shown as the mean of the group in panel A and those from experiment 3 in panel C. Red lines indicate non-immune pigs and black lines indicate immune pigs. Viraemia estimated by qPCR for individual pigs in experiments 1 and 3 are shown in panels B and D respectively and expressed as ASFV genome copy number per ml blood (log_10_). (For interpretation of the references to colour in this figure legend, the reader is referred to the web version of this article.)

**Fig. 3:**
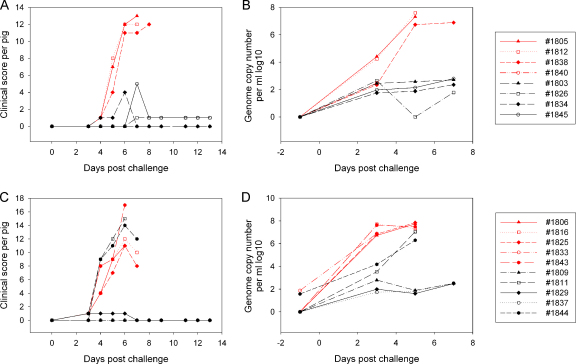
Clinical score (A and C) and viraemia estimated by qPCR (B and D) of individual pigs from experiment 2. Results from immune pigs challenged with virulent Uganda 1965 (A and B), or Benin 97/1 (C and D) isolates are shown in black lines. Results from non-immune control pigs challenged with Benin 97/1 or Uganda 1965 are shown as red lines. Two immune pigs (#1811: black circle; #1844: black square) were not protected from Benin 97/1 challenge (C and D).

All 9 immune pigs from experiments 1 and 3 were protected from challenge with the Benin 97/1 without any clinical signs of ASF (Figs. 1 and 2). In experiment 2, the 4 immune pigs challenged with the virulent Uganda 1965 isolate were all protected, although 2 of these pigs showed very short transient pyrexia. However, 2 pigs (1811, 1844) from experiment 2 were not protected following challenge with Benin 97/1 (Figs. 1 and 3). Thus the survival rate of immune pigs challenged with either Benin 97/1 or Uganda 1965 virulent isolates was 100% in two experiments (Figs. 1 and 3) and 60% following challenge with Benin 97/1 in experiment 2. In experiment 1, no adverse effects or clinical signs were observed following the immunisation, the boost or challenge. In one pig (VR89) low copy numbers of virus genome were detected in blood by qPCR, but not by HAD assay, at 14 days post-boost with OURT88/1 (data not shown). ASFV was not detected in any tissues collected from immune pigs at the termination of the experiment. In contrast, all the non-immune pigs challenged with Benin 97/1, developed typical ASF symptoms including high viraemia (∼10^7^ copies of the virus genome/ml; and up to 8.8 HAD_50_/ml virus), and died or were euthanized for ethical reasons within 7 days of challenge (Fig. 2A and B). Post-mortem examination and detection of ASFV from tissues collected from these animals by qPCR and HAD assay confirmed severe ASFV infection in the non-immune pigs (up to 10^7^ HAD_50_/mg tissue) (see summary in Supplementary Table 2).

In the second experiment of the 12 immunised pigs, 5 (pig numbers 1826, 1829, 1834, 1837 and 1845) developed a transient pyrexia (Supplementary Fig. 1) following immunisation with OURT88/3. After the OURT88/1 boost, 4 pigs (pig numbers 1809, 1819, 1822 and 1841) developed pyrexia (Supplementary Fig. 1). Viraemia was detected from pigs 1819 and 1841 by qPCR and HAD assays (4.07 × 10^6^ genome copies/ml: 6 HAD_50_/ml and 6.19 × 10^3^ genome copies/ml: 3.25 HAD_50_/ml respectively). Virus genome was detected at low copy numbers by qPCR in blood samples from an additional 2 pigs but these were negative by HAD assay. Pigs 1819, 1822 and 1841 were terminated for ethical reasons between day 4 and day 6 post boost with OURT88/1 before the potential development of severe ASF symptoms.

Because of the loss of pigs after the OURT88/1 boost, only four pigs were subsequently challenged with virulent Uganda 1965. Two of these developed transient pyrexia and low viraemia. Pig 1834 had a temperature at day 6 of 40.3 °C, and the virus genome was detected at 227 copies/ml and virus at 1.75 HAD_50_/ml; pig 1845 had a temperature at day 7 of 40.6 °C and the virus genome was detected at 633 copies/ml; and virus at 2 HAD_50_/ml. The other two pigs challenged with virulent Uganda 1965 isolate showed no clinical signs and no virus was detected in blood by qPCR or HAD assay. Five pigs were challenged with Benin 97/1, two pigs (1811, 1844) developed typical ASF (Fig. 3C and D) and were terminated at days 6 and 7 respectively before developing severe disease. The remaining pigs (1809, 1829, 1837) did not develop pyrexia or other ASF clinical signs but occasionally virus genome was detected by qPCR at concentrations up to 323 copies/ml but virus was not detected by HAD assay.

The two groups of naïve pigs challenged with either virulent Uganda 1965 or Benin 97/1 all developed severe clinical signs of ASF with high viraemia (up to 5.37 × 10^7^ genome copies/ml; virus up to 7.25 HAD_50_), and either died or were terminated within 8 days of challenge (Fig. 3). Post-mortem examination confirmed severe ASF in these control pigs (see summary in Supplementary Table 2).

In the third experiment, 7 immune pigs were generated and 6 of these were challenged with Benin 97/1. One pig (474) showed pyrexia from 2 weeks after the first immunisation (Supplementary Fig. 1C). This pig was euthanised before the OURT88/1 boost. Post-mortem examination of this pig revealed a dark enlarged spleen characteristic of ASFV infection and virus DNA was detected from the spleen and retropharyngeal lymph node (RLN) by qPCR (8790 and 41000 virus genome copies/mg tissue respectively) and by cytopathic effect in cultures of porcine macrophages. HAD was not observed in these cultures, indicating that the replicating virus was non-HAD, as expected for the OURT88/3 isolate. Six pigs each of the immune and non-immune groups were challenged with Benin 97/1. All of the immunised pigs were protected from challenge without showing any clinical signs or development of viraemia (Fig. 2C and D). Low copy numbers of the virus genome were detected by qPCR, but not HAD, in spleen and RLN of pig 55 at the termination of the experiment but not in any other lymphoid tissues and blood in this pig, or in any tissues from the other immunised and challenged pigs. In contrast, high copy numbers of virus genome and of virus were detected in blood (up to 5.62 × 10^8^ virus genome copies/ml; virus up to 8.3 HAD_50_/ml) and tissues (virus ∼7 HAD_50_/mg of tissue) were detected from all lymphoid tissues in all of the non-immune pigs challenged (see summary in Supplementary Table 2).

Unlike the non-immune pigs, immune pigs challenged increased their body weight during the challenge (Supplementary Fig. 2).

### 3.2 Measurement of ASFV specific T cell and antibody responses in immunised pigs

**Fig. 4:**
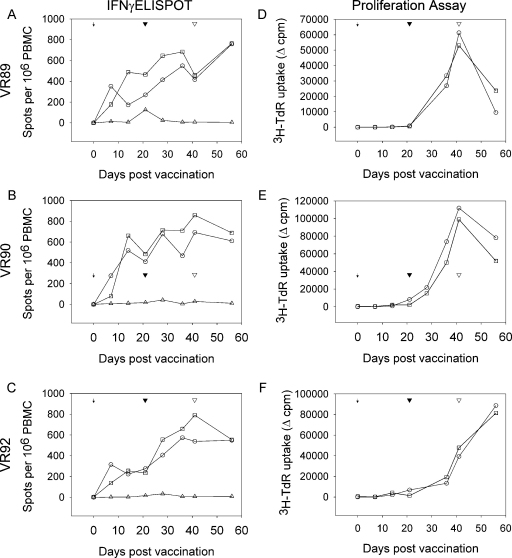
Development of anti-ASFV T cell responses after OURT88/3 immunisation, assessed by IFN-γ ELISPOT (A–C) and proliferation assays (D–F) from experiment 1. Pig peripheral blood lymphocytes were stimulated *ex vivo* with either OURT88/3 (open circle) or Benin 97/1 (open square). Background levels of the ELISPOT assays are shown in black open triangles. ELISPOT results are shown as IFN-γ production per one million lymphocytes, and proliferation assays are displayed as [^3^H] thymidine uptake (Δcpm [experimental cpm − BG cpm]). The *x*-axis shows days post the first ASFV inoculation. The arrow on each graph indicates the time of OURT88/3 immunisation, the black arrowhead indicates the time of OURT88/1 boost and the open arrowhead indicates the Benin challenge.

Lymphocytes from immunised pigs in experiment 1 were collected at various times post-immunisation and IFN-γ ELISPOT and proliferation assays were performed with OURT88/3 or Benin 97/1 as antigen. In all 3 pigs, the numbers of ASFV specific IFN-γ producing cells was rapidly increased after the OURT88/3 inoculation and further increased after the OURT88/1 boost. Both OURT88/3 and Benin 97/1 isolates stimulated lymphocytes from immunised pigs to an approximately equal amount (Fig. 4A–C). Low levels of proliferation were detected in all pigs at 1 or 2 weeks post-OURT88/3 inoculation, but the amount of proliferation was dramatically increased after the OURT88/1 boost (Fig. 4D–F). In two of the pigs (Fig. 4D and E) levels of T cell proliferative responses dropped following challenge with Benin 97/1 isolate and in the other pig levels continued to rise (Fig. 4F).

**Fig. 5:**
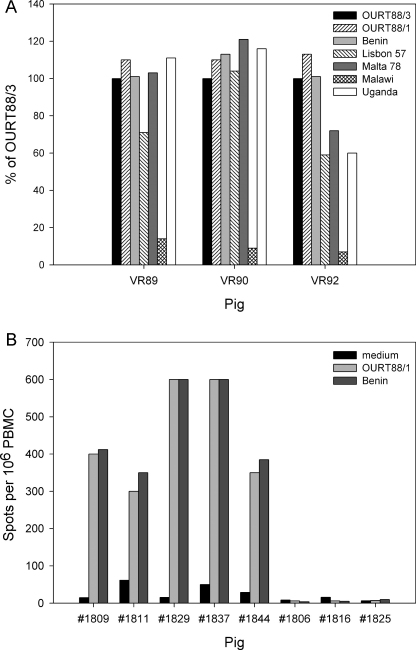
ASFV isolate cross-reactivity measured by IFN-γ ELISPOT assays. The pigs from experiment 1 (VR89, VR90, VR92) which were protected from challenge with Benin 97/1 isolate were used as immune lymphocytes donors for the *ex vivo* IFN-γ ELISPOT assay following stimulation of the lymphocytes with various ASFV isolates. Results are shown as % cross-reactivity compared to the OURT88/3 stimulation. Panel B shows the stimulation of lymphocytes from pigs in experiment 2. Immunised pigs (1809, 1811, 1829, 1837, 1844) and non-immune control pigs (1806, 1816, 1825) peripheral blood mononuclear cells collected a day before challenge were stimulated *ex vivo* with either medium alone, OURT88/1 or Benin 97/1. Results are shown as IFN-γ production per one million lymphocytes. The *x*-axis shows the pig number.

At the termination of the experiment, lymphocytes from these pigs were tested for cross-reactivity stimulated with various ASFV isolates by IFN-γ ELISPOT assays (Fig. 5A). Immune lymphocytes from all 3 pigs responded similarly to OURT88/3, OURT88/1 and Benin 97/1. Lymphocytes from two pigs (VR89, VR90) also responded well to genotype 1 isolate Malta 78 and genotype X isolate Uganda 1965 and lymphocytes from pig VR90 also responded well to genotype I isolate Lisbon 57. Lymphocytes from pig VR92 responded less well to Malta 78, Uganda 1965 and Lisbon 57 and those from pig VR89 also showed a reduced response to Lisbon 57. No cross-reactivity was observed to genotype VIII isolate Malawi Lil 20/1.

In the second experiment (Fig. 5B), lymphocytes were collected from pigs just prior to challenge. Lymphocytes from 2 of the immunised pigs (1829, 1837) showed a much stronger response in IFN-γ ELISPOT assays against OURT88/1 and Benin 97/1 than the other 3 immunised pigs (1809, 1811, 1844). Interestingly, 2 of the pigs from which lymphocytes responded least (1811, 1844) in IFN-γ ELISPOT assays (Fig. 5B) were those which were not protected against Benin 97/1 challenge (Fig. 3C and D). No response was observed in IFN-γ ELISPOT assays when lymphocytes from non-immune pigs 1806, 1816, 1825 (Fig. 5B) were stimulated with ASFV, confirming the specificity of the assay. In the third experiment IFN-γ ELISPOT assay was carried out using lymphocytes collected prior to challenge and the results were too high to be read accurately by the ELISPOT reader (data not shown). This indicates that strong T cell immunity was induced in all pigs before the challenge.

**Fig. 6:**
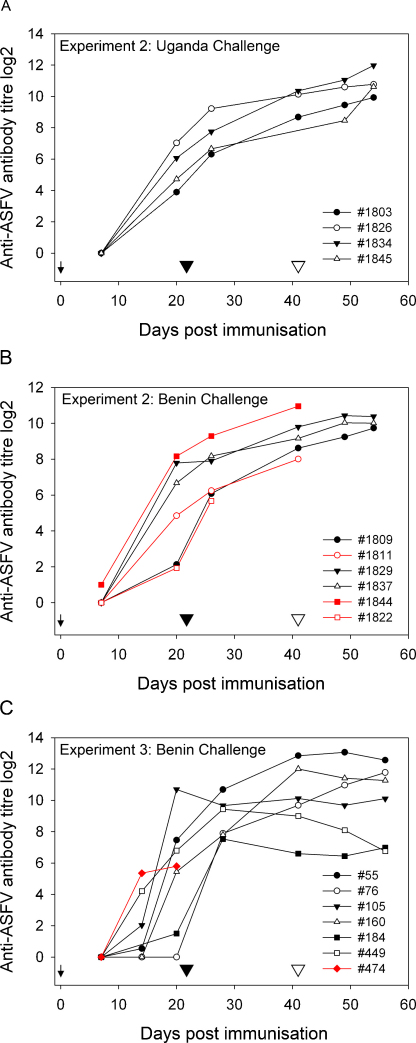
Anti-ASFV VP72 antibody responses following immunisation from experiment 2 (A and B) and experiment 3 (C). Antibody titre was measured by competitive ELISA in serial dilution (log_2_) giving 50% inhibition. Groups of pigs from experiment 2 challenged with Uganda 1965 are shown in A and challenged with Benin 97/1 in B. A group of pigs from experiment 3 challenged with Benin 97/1 is shown in C. The arrow on each graph indicates the time of OURT88/3 immunisation, the black arrowhead indicates the time of OURT88/1 boost and the open arrowhead indicates the challenge. Red line/symbol indicates pigs not protected from challenge and/or lost at immunisation procedures.

A competitive ELISA based on the p72 major capsid protein was used to measure development of anti-ASFV specific antibodies. The results from analysis of sera collected in experiment 2 and 3 are shown in Fig. 6. An antibody response developed in all pigs immunised with OURT88/3 followed by OURT88/1 boost, except pig 76 from experiment 3 in which antibody against p72 was not detected prior to boost (Fig. 6C). The levels of anti-ASFV antibody gradually increased and were boosted by the OURT88/1 inoculation. Interestingly, the antibody levels in the 2 pigs which were not protected from Benin 97/1 challenge in experiment 2 (Fig. 6B) had either the highest (1844) or the lowest (1811) anti-ASFV antibody titre before the challenge. On the other hand pig 184 from experiment 3 had a much lower antibody titre at challenge (day 41) than these unprotected pigs in experiment 2, but was protected. The pig which was euthanized following boost (1822) had the lowest antibody titres at the time of boost (Fig. 6B), in contrast pig 76 from experiment 3 was protected from OURT88/1 boost despite a lack of apparent antibody response (Fig. 6C).

## 4 Discussion

In this study we have demonstrated that experimental immunisation of pigs with a non-virulent ASFV genotype I isolate from Portugal, OURT88/3, followed by a boost with a closely related virulent isolate, OURT88/1, can induce protective immunity in European domestic pigs against challenge from two virulent African isolates of ASFV. These included a genotype I isolate from West Africa, Benin 97/1 and a genotype X isolate from Uganda, virulent Uganda 1965. Overall 85.7% and 100% pigs were protected from Benin 97/1 and Uganda 1965 ASFV challenge respectively. More than 78% of pigs challenged with Benin 97/1 and 50% of pigs challenged with Uganda 1965 were completely protected by not showing any sign of disease or development of viraemia.

Phylogenetic analysis of the concatenated sequences of 125 genes conserved between 12 complete genome sequences showed that the OURT88/3 and Benin 97/1 sequences are greater than 95% identical across these genes [15,16]. Although the virulent Uganda 1965 isolate is placed in VP72 genotype X, it falls within the same clade as the genotype I isolates (Chapman et al., unpublished observations). This is the first clear demonstration of induction of cross-protective immunity against challenge with more distantly related virulent strains of ASFV. It has been reported previously that the pigs which recover from less virulent strains of ASFV are resistant to challenge with the same or very closely related virus strains [1,3,14]. The genotypes of the strains used in these studies were not defined.

The ASFV OURT88/3 strain was isolated from *Ornithodoros erraticus* ticks in Portugal and described not to cause clinical signs or viraemia [2]. Interestingly, the inoculation of virulent OURT88/1 virus following OURT88/3 immunisation, could protect pigs from the disease, and also further stimulated development of anti-ASFV immune responses. This indicates that the inoculation of OURT88/1 acts to boost the immune response (Figs. 4 and 6) and this might be required for inducing sufficient ASFV isolate-cross-protective immunity. However, further experiments are required to clarify this. Measurement of ASFV specific IFN-γ responses *ex vivo*, with different ASFV isolates, showed various degrees of cross-reactivity and this correlated well with cross-protection induced *in vivo*. Good cross-reactivity against genotype X isolate virulent Uganda 1965 (Fig. 5A) was observed, and this is the reason why pigs were challenged with virulent Uganda 1965 in experiment 2. As predicted from this *ex vivo* assay, all of the pigs immunised and challenged with virulent Uganda 1965 virus were protected. No cross-reactivity to genotype XIII isolate Malawi LIL 20/1 was detected and this correlates with the observation that OURT88/3 and OURT88/1 immunised pigs are not protected from Malawi LIL 20/1 challenge [2,Denyer et al. unpublished observation]. Taken together these data suggest that this *ex vivo*, IFN-γ ELISPOT assay might be a useful tool to assess vaccine efficacy and/or to assess possibility of ASFV isolate-cross-protection.

An anti-ASFV antibody response also developed after OURT88/3 immunisation and was boosted after the OURT88/1 inoculation. The anti-ASFV antibody titre was measured by a p72 competition ELISA, however we could not conclude from these experiments whether the level of antibody developed by our immunisation protocol is either sufficient or necessary for protection.

OURT88/3 has been used as a vaccine model to identify what is required for inducing ASFV protective immunity in domestic pigs. The observations of adverse effects of OURT88/3 immunisation in some of the pigs vaccinated in France suggest that further attenuation of this isolate by deleting additional genes or possibly changing the dose or route of vaccination may be useful. Secondly, the results from experiment 2 showed that our current protocol did not induce complete protection in all of the pigs immunised with the virulent OURT88/1 boost. This may be due to the genetic background of the pigs as we have previously demonstrated that cc inbred pigs are also not always protected by OURT88/3 from OURT88/1 challenge [11]. It is possible that the age and/or size of pigs at the time of the first immunisation may be important for the induction of complete protection since the pigs used in France were smaller and younger than those used at Pirbright. It will also be useful in future to compare the effects of boosting with the non or low virulent OURT88/3 since this would help to avoid adverse effects resulting from boosting with virulent OURT88/1. Our observation that cross-protection can be induced between different genotypes is important since this suggests when an ASFV vaccine is developed, its practical use in the field is likely to be extended in areas where several genotypes are present. Additional experiments are required to establish the extent of cross-protection.
