# Supplementary material for: Protection of European domestic pigs from virulent African isolates of African swine fever virus by experimental immunisation
Source: Vaccine. 2011 Jun 20;29(28):4593–600. doi: 10.1016/j.vaccine.2011.04.052 (PMC3120964; doi:10.1016/j.vaccine.2011.04.052)
Supplement: Supplementary file 1 [file mmc1.pdf]

Pyrexia after immunisation of Large White pigs with OUR T88/3 in experiment 2

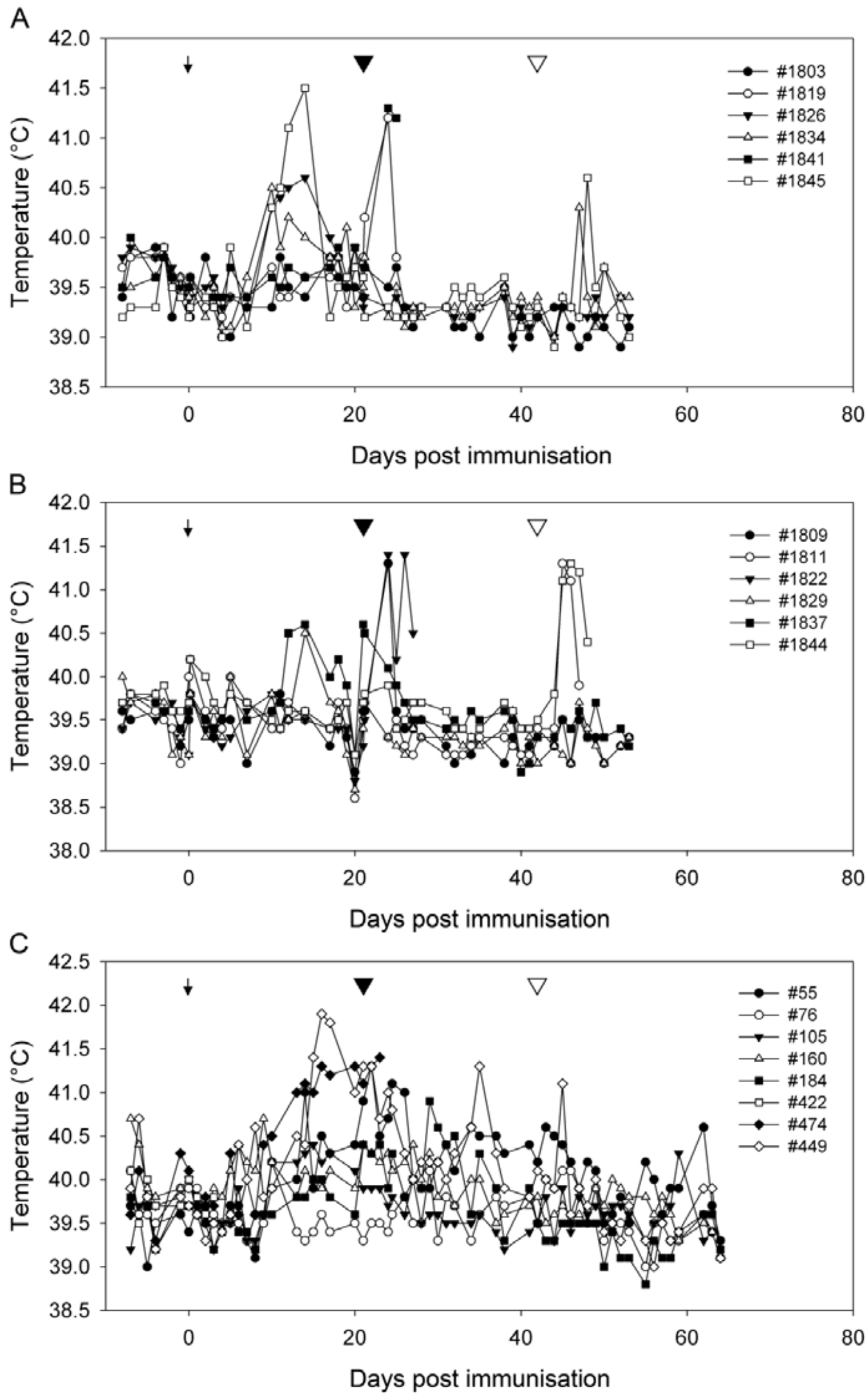

Supplementary figure 1. Pyrexia was observed in some pigs during experiments 2 and 3 after immunisation. Body temperatures are shown on the y-axis and the time post inoculation with OURT88/3 is shown in days. Panel A shows temperatures of immune pigs from experiment 2 that were challenged with Uganda, panel B shows those challenged with Benin. Panel C shows temperatures of immune pigs from experiment 3 that were challenged with Benin. The arrow on each graph indicates the time of OURT88/3 immunisation, the black arrowhead indicates the time of OURT88/1 boost and the open arrowhead indicates the challenge.

Immune pigs continue to put on weight after challenge with virulent African swine fever virus

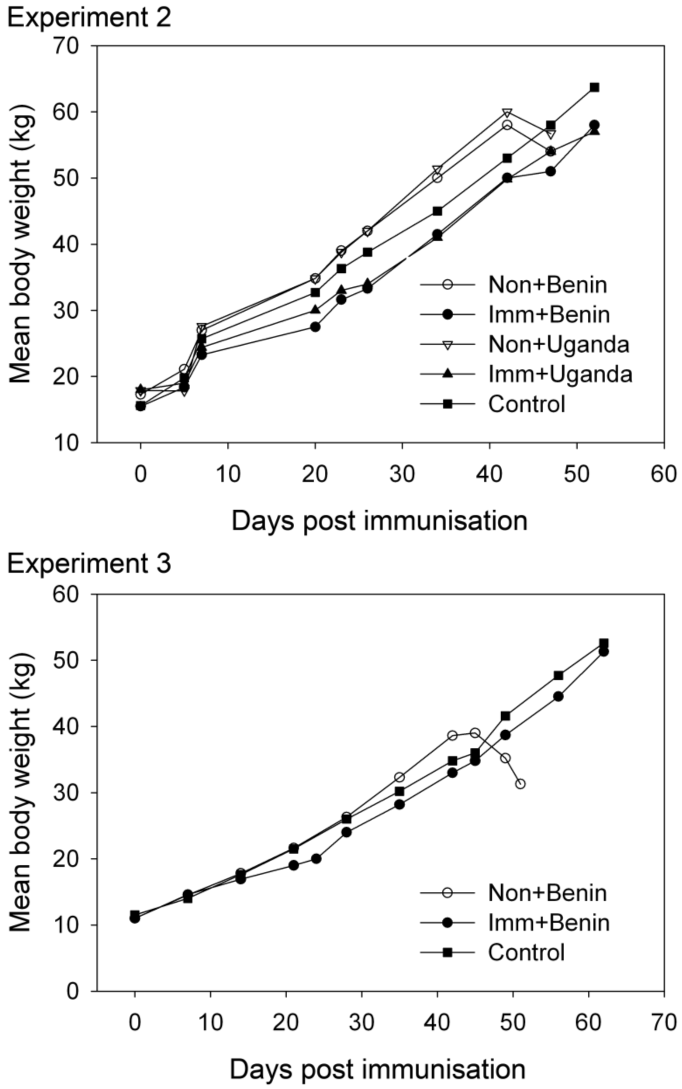

Supplementary figure 2. Mean weight per group from experiments 2 and 3 over time. Non-immune pig groups challenged with virulent ASFV are labelled as Non+ and immune pigs as Imm+, with the virus isolate indicated in each case.

Clinical scoring system for African swine fever

Supplementary Table 1 The guideline of clinical score for ASF*

1.Temperature :<39 = 0, 39.0 < to < 39.5= 1, 39.5 ≤ to < 40 = 2, 40.0 to ≤ 40.5 = 3, 40.6 ≤ 41 = 4, >41 = 5
2. Inappitence : Reduced eating (1), Only picking at food (4), Not eating (6)
3. Recumbancy : Lethargic (1), Get up only when touched (2), Slow to get up when touched (4), Remain ecumbent when touched (6)
4. Skin Haemorrhage : Haemorrhagic areas on ears and body (1), Generalised haemorrhage all over body (3)
5. Joint Swelling : a joint swelling (1), Severe swelling with difficulty walking (4)
5. Laboured breathing and/or coughing (1), Severe (3)
7. Occular discharge (1),(gummed up eyes)
8. Diarrhoea (1), Bloody Diarrhoea (4)
9. Blood in Urine (4)
10. Vomiting (4)

*The clinical assessment of ASFV was composed of 10 different categories each scored between 0 and 6.

Supplementary Table 2. Summary of pig numbers for immunised, challenged, shown symptoms, death, virus positive by PCR and HAD

	Inoculation 1		Inoculation 2		Challenge		Number of viraemic, survived pigs		Virus detection in tissues from survived pigs at termination	
	Symptoms	Death	Symptoms	Death	Symptoms	Death†	PCR	HAD	PCR	HAD
EXP 1	OURT88/3		OURT88/1		Benin97					
Non-immune	-	-	-	-	4/4	4/4	-	-	-	-
Immunised	0/3	0/3	0/3	0/3	0/3	0/3	0/3	0/3	0/3	0/3
EXP 2-A	OURT88/3		OURT88/1		Uganda					
Non-immune	-	-	-	-	5/5	5/5	-	-	-	-
Immunised	3/6	0/6	2/6	2/6	2/4	0/4	2/4	2/4	NT	NT
EXP 2-B	OURT88/3		OURT88/1		Benin 97					
Non-immune	-	-	-	-	5/5	5/5	-	-	-	-
Immunised	2/6	0/6	2/6	1/6	2/5	2/5	3/3	0/3	NT	NT
EXP 3	OURT88/3		OURT88/1		Benin 97					
Non-immune	-	-	-	-	6/6	6/6	-	-	-	-
Immunised	1/7	1/7	0/6	0/6	0/6	0/6	0/6	0/6	1/6	0/6

†: All dead pigs were positive for viraemia and infectious ASFV in tissues by both PCR and HAD

NT: Not tested
